# Comparison of Atypical and Osteoporotic Femoral Shaft Fractures in the Elderly: A Multicenter Study

**DOI:** 10.1155/2018/1068053

**Published:** 2018-05-16

**Authors:** Takanori Miura, Hiroaki Kijima, Noriyuki Ishikawa, Toshihito Ebina, Takayuki Tani, Shuichi Chida, Tetsuya Suzuki, Satoshi Yumto, Hiroshi Tazawa, Naohisa Miyakoshi, Yoichi Shimada

**Affiliations:** ^1^Department of Orthopedic Surgery, Kakunodate General Hospital, 3-Iwase Kakunodatemachi, Semboku, Akita 014-0394, Japan; ^2^Department of Orthopedic Surgery, Akita University Graduate School of Medicine, 1-1-1 Hondo, Akita 010-8543, Japan; ^3^Department of Orthopedic Surgery, Akita Red Cross Hospital, Kamikitate Saruta, Aza-Naeshirosawa 222-1, Akita 010-1406, Japan

## Abstract

**Background:**

In atypical femoral fractures, owing to the high rates of complications and delayed healing that accompany the plate fixation, the most favorable treatment is intramedullary nailing. Although there is insufficient evidence, plate fixation is chosen due to anterolateral bowing of the femur. This study compared the bone healing time and rates of complications in atypical femoral shaft fractures and osteoporotic femoral shaft fractures.

**Methods:**

We searched the medical records of 3 institutions in Japan for patients with femoral shaft fractures who visited between 1 January 2010 and 31 December 2015. We identified 65 patients and excluded 37 among these due to high-energy injuries or being younger than 65 years. Among the remaining patients, we identified 17 and 11 women with atypical (AFF group) and osteoporotic femoral shaft fractures (OP group), respectively.

**Results:**

In surgical method, there were differences in intramedullary nailing (94.1% versus 27.2%) (*p* < 0.01). The mean bone healing time was 11.1 months versus 6.7 months in 2 groups (*p* < 0.01). Iatrogenic femoral fractures during intramedullary nail insertion were observed in both groups, and reoperation was only seen in atypical femoral fractures treated with a plate fixation, but there was no difference in the rate of complications (23.5% versus 9.1%).

**Conclusions:**

In the atypical femoral fracture group, intramedullary nailing was more chosen, but the bone healing time was delayed and plate fixation cases needed reoperation. There was no significant difference in the rate of complications between the 2 groups.

## 1. Introduction

Atypical femoral fracture (AFF) is a fracture that occurs at the femoral subtrochanteric or shaft region. The occurrence of this type of fracture is associated with the long-term use of bisphosphonates (BPs) and bowed femoral shaft stress fractures [1–4]. It is a common conception that atypical femoral shaft fractures heal poorly [1, 5], but there have been few reports comparing patients with AFF and the control group with respect to bone healing times [5]. Among the treatment options, intramedullary nail fixation is most favorable. But in some cases with a high degree of lateral femoral bowing, it is difficult to insert the intramedullary nail, and there are consequently high rates of intraoperative fractures and implant failure during the procedure in patients with AFF [6, 7]. However, the sample size available for the examination of AFF in a single institution is limited. The present multicenter study aimed to compare the bone healing time and rates of complications in patients with atypical femoral shaft fractures and those with osteoporotic femoral shaft fractures.

## 2. Materials and Methods

### 2.1. Inclusion Criteria

To identify patients with AFF of the femoral shaft and osteoporotic femoral shaft fractures, we reviewed digitized radiographs of all patients, from 3 institutions, who had sustained fractures of the femoral shaft and received acute treatment for them between 1 January 2010 and 31 December 2015. Patient medical and drug treatment histories were obtained from medical records. Patients with primary or secondary metabolic disorders of the skeleton apart from osteoporosis were excluded. Patients with AFF were identified by the radiographic pattern consisting of a transverse fracture line on the lateral side of the femoral shaft with focal thickening (callus reaction) around the fracture and no or minimal comminution, in accordance with the American Society for Bone and Mineral Research Task Force's major criteria, 2nd version [1]. Osteoporotic fractures were defined as spiral, oblique, or comminuted shaft fractures below the lesser trochanter and above the supracondylar flare, and this definition included periprosthetic fractures.

### 2.2. Study Patients

We identified 65 patients; however, we excluded 37 patients who had high-energy injuries or who were below 65 years of age. The remaining 28 patients were all above 65 years of age and sustained low-energy-mechanism injuries, such as a fall from standing height or lower. All patients with fractures were routinely asked by the admitting physician whether they were taking or had a history of taking BPs.

The mean follow-up time for patients with atypical fractures and osteoporotic femoral fractures was 17.26 months (range: 6–36 months) and 10.27 months (range: 3–28 months), respectively. No patients were lost to follow-up. No patients were recalled specifically for this study; all data were obtained from medical records or radiographs. Healing was judged by 2 observers using the following criteria: the ability to fully bear weight, lack of pain at the fracture site, and radiographic consolidation as observed on orthogonal plain radiographic views. All patients included in the current study had all clinical and radiographic data available for analysis. We determined differences in the continuous variables of interest (age and bone healing time) between the AFF (AFF group) and osteoporotic fractures group (OP group). The overall data were examined using descriptive statistics, with frequencies given for categorical variables and means and standard deviations for continuous variables. IBM SPSS Statistics Version V22.0 was used for all analyses.

## 3. Results

The demographic data are shown in Table 1. All patients were women; 17 patients had complete atypical femoral shaft fractures [average age: 80.7 years (range: 77–88 years)], and the remaining 11 had osteoporotic femoral shaft fractures [average age: 81.0 years (range: 65–96 years)]. There were no significant differences in age between the two groups (*p* = 0.67). The patients with AFF used BPs more often (15 patients, 88%) compared to those with osteoporotic femoral fractures (2 patients, 18%) (*p* < 0.01). For treatment, all patients with atypical fractures and 81.8% (9 of 11) with osteoporotic fractures underwent surgery (*p* = 0.40). In the group with AFF, intramedullary nail fixation was performed in 94.1% (16 of 17) and plate fixation in 5.9% (1 of 17). In the group with osteoporotic femoral fractures, intramedullary nail fixation was performed in 27.2% (3 of 11), plate fixation in 36.3% (4 of 11), replacement arthroplasty in 18.2% (2 of 11), and conservative treatment in 18.2% (2 of 11). Intramedullary nail fixation was used more frequently in the group with atypical femoral fractures (94.1% versus 27.2%; *p* < 0.01). Teriparatide was used in 41.2% (7 patients) with AFF and in 18.2% (2 patients) with osteoporotic femoral fractures (*p* = 0.25). Low-intensity pulsed ultrasound (LIPUS) therapy was performed postoperatively in 23.5% (4 patients) in the group with AFF and in 9.1% (1 patient) in the group with osteoporotic femoral fractures (*p* = 0.30).

**Figure 1:**
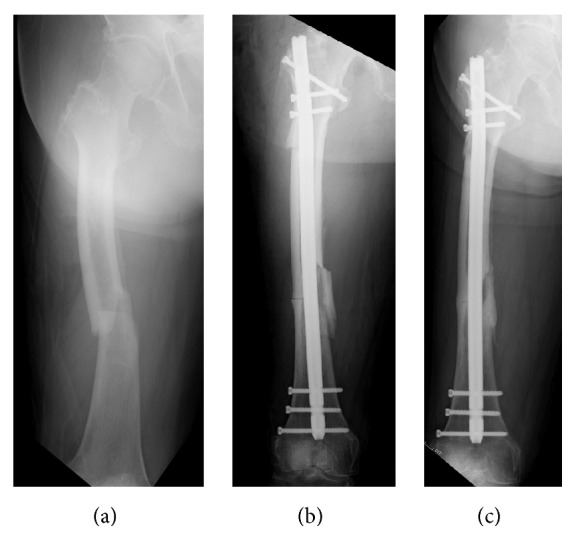
(a) AP radiographs of a patient with atypical femoral shaft fracture (b) treated with an intramedullary nail and intraoperative peri-implant fracture occurred. (c) Bone healed after 32 weeks from the index surgery.

**Figure 2:**
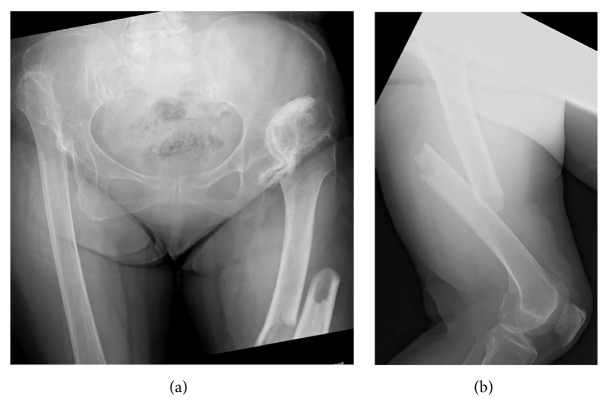
(a) AP and (b) lateral radiographs show a characteristic atypical femoral fracture and severe osteoarthritis of the hip.

**Figure 3:**
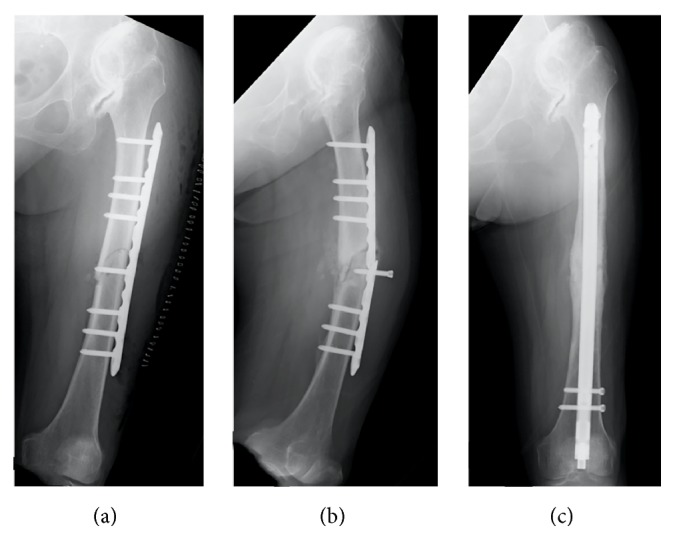
(a) AP image is shown, which was obtained after open reduction and internal fixation with an LCP plate. (b) AP radiographs obtained 22 weeks after fracture surgery show plate breakage. (c) AP image obtained 43 weeks after initial fracture surgery; reoperation was performed with retrograde intramedullary nailing.

The main results are shown in Table 2. We determined the mean bone healing time in the group with AFF to be 11.1 months (range: 5–28 months; SD: 6.1 months) and median bone healing time to be 10 months (6–14.5 months; interquartile range; 25–75 ); in the group with osteoporotic femoral fractures, we determined the mean bone healing time to be 6.7 months (range: 2.5–28 months; SD: 8.1 months) and median bone healing time to be 3.25 months (2.75–5.5 months; interquartile range: 25–75). This was a significant difference between the two groups (*p* < 0.01). There were no statistically significant differences in the rates of complications in the two groups (23.5% versus 9.1%; *p* = 0.62). In the group with AFF, intraoperative femoral fracture during intramedullary nail insertion occurred in 11.8% (2 cases) (Figure 1), and one plate failure occurred (5.9%); there were no nail failures. In the group with osteoporotic fractures, there was only 1 case of intraoperative fracture (9.1%) and no implant failures. There was 1 reoperation among the 17 patients with AFF, which was treated with BPs and fixation with a plate. There were no reoperations in the 11 patients with osteoporotic femoral shaft fractures. One patient had osteoarthritis of the hip, and the deformity was severe (Figure 2). We considered that antegrade intramedullary nail insertion would be difficult and performed plate fixation. But implant failure occurred, and retrograde nail fixation was performed 22 weeks after the index surgery. The time to union was 43 weeks after the first surgery (Figure 3). The patient reports having no pain and can walk with a cane.

## 4. Discussion

The present study demonstrated a statistically significant difference in the bone healing time between patients with atypical femoral shaft fractures and those with osteoporotic femoral shaft fractures (11.1 months versus 6.7 months, *p* < 0.01). However, the rate of complications was 23.5% (AFF) versus 9.1% (osteoporotic femoral shaft fractures) for the two groups, which was not significant, although we experienced 1 plate failure and required revision surgery with intramedullary nail fixation in the AFF group.

There have been reports showing that the long-term use of BPs, steroids, rheumatoid arthritis, femoral lateral bowing, and diabetes mellitus are associated with the risk of AFF [1, 4, 8–12]. A multifactorial etiology, including poor bone quality due to mutual interactions and mechanical stress, appears to be responsible for the occurrence of AFF [4, 6]. In particular, patients with severe lateral bowing of the femur are likely to sustain dynamic stress on the femoral shaft due to axial pressure [4]; as a result, femoral shat fractures are increased [2–4]. In surgical treatment, intramedullary nail fixation has been recommended [2], and plates were previously chosen as the fixation device owing to the technical impracticability of nailing due to bowing of the femur or cortex thickening [7]. However, in atypical femoral shaft fractures, fragility of the bone, lateral bowing of the femur, and varus moment arm are associated with high rates of complications [5, 7]. Lateral bowing of the femur was associated with difficulties in nail insertion during the operation and iatrogenic fracturing during nail placement [5]. Delayed bone healing was associated with postoperative plate failure [7]. In the plate fixation procedure in particular, complications rates were high and outcomes poor [6, 7]. So, choosing the fixation material in atypical femoral shaft fractures is difficult.

First, this analysis revealed that atypical femoral shaft fractures delay bone healing. However, there were statistical differences in the rate of use of intramedullary nails; it took an average of 11.1 months until bone healing with AFF was considered to be a prominent feature of AFF. In the osteoporotic femoral shaft fractures group, due to existing implants, plate fixation was more chosen. Second, the rate of complications was 23.5% (AFF) versus 9.1% (osteoporotic femur fracture) between the two groups, which was not significant. Although we experienced 1 plate failure and required revision surgery with intramedullary nail fixation in the AFF patient, only 1 patient underwent plate fixation in the AFF group. But the 4 patients who underwent plate fixation in the osteoporotic femoral fractures group had no complications, and their bone healing was good. The use of plates rather than intramedullary fixation devices might have contributed to the high reoperation rate observed elsewhere [5]. In patients with AFF, intramedullary nail insertion should be the first choice in treatment. However, there are some cases in which antegrade intramedullary nail insertion will be difficult. In our case, retrograde nail insertion was useful for severe hip osteoarthritis.

We used a similar array of implants in both groups, although more adjuvants tended to be given to patients receiving antiresorptive therapy. In particular, more teriparatide was given postoperatively to patients in the AFF group. The use of teriparatide in patients with AFF has been reported to significantly shorten the postoperative recovery and reduce rates of delayed healing or nonunion [13]. And its efficacy in AFF has been proposed in a systematic review [14]. Thus, the use of teriparatide may be useful for the treatment of AFF.

Our study has some limitations. We had a limited number of Japanese patients. It is difficult to obtain a large sample in the study of a relatively rare injury. Complications were rare, and there were no significant differences between the two groups, and there is a possibility that this outcome differs from other races. Retrospective studies carry an inherent risk of observer bias, including the potential for missing data and an inability to control for confounding variables. We did not obtain functional outcomes for the patients, and we were unable to rigorously evaluate and compare the time to recovery between the two groups. It is worthwhile to note that, for this study, few similar correlational studies have been attempted; therefore, potential for further exploration is substantial. Future research on this topic should include a larger sample size and stratify patients according to different ethnicities if the sample size permits.

## 5. Conclusion

Atypical femoral shaft fractures are often seen in those treated with antiresorptive therapy; in this study, intramedullary nailing was chosen more often, but the time to bone healing was delayed compared with osteoporotic femoral fractures. There was no significant difference in complications rates, but iatrogenic femoral fractures occurred during intramedullary nail insertion, and plate fixation cases needed reoperation. Therefore, we should distinguish atypical femoral shaft fractures from osteoporotic femoral shaft fractures.

## Figures and Tables

**Table 1:** Patient characteristics and treatments.

Parameter	AFF (*n* = 17)	OP (*n* = 11)	*p*	Test
Male : Female	0 : 17	0 : 11	—	—
Age (years)	80.7 (77–88)	81.0 (65–96)	0.67	Mann–Whitney *U* test
Antiresorptive therapy (%)	88.2	18.2	<0.01	Fisher's exact test
Surgery (%)	100	81.8	0.40	Fisher's exact test
Intramedullary nail (%)	94.1	27.2	<0.01	Fisher's exact test
Fixation with plate (%)	5.9	36.3	0.06	Fisher's exact test
Replacement arthroplasty (%)	0	18.2	0.15	Fisher's exact test
Conservative treatment (%)	0	18.2	0.15	Fisher's exact test
Teriparatide treatment (%)	41.2	18.2	0.25	Fisher's exact test
Postoperative LIPUS treatment (%)	23.5	9.1	0.30	Fisher's exact test

**Table 2:** Comparison of bone healing time and complication rates.

Parameter	AFF (*n* = 17)	OP (*n* = 11)	*p*	test
Bone healing time (months)	11.1 (5–28)	6.7 (2.5−28)	<0.01	Mann–Whitney *U* test
Complication (%)	23.5	9.1	0.62	Fisher's exact test
Intraoperative fracture (%)	11.8	9.1	0.66	Fisher's exact test
Implant failure (%)	5.9	0	0.61	Fisher's exact test
Reoperation (%)	5.9	0	0.61	Fisher's exact test
